# Data-driven approach to implementation mapping for the selection of implementation strategies: a case example for risk-aligned bladder cancer surveillance

**DOI:** 10.1186/s13012-022-01231-6

**Published:** 2022-09-01

**Authors:** Florian R. Schroeck, A. Aziz Ould Ismail, David A. Haggstrom, Steven L. Sanchez, DeRon R. Walker, Lisa Zubkoff

**Affiliations:** 1 White River Junction VA Medical Center, White River Junction, VT USA; 2 Section of Urology, Dartmouth Hitchcock Medical Center, Lebanon, NH USA; 3 Dartmouth Cancer Center, Lebanon, NH USA; 4 The Dartmouth Institute for Health Policy and Clinical Practice, Geisel School of Medicine at Dartmouth College, Lebanon, USA; 5 VA HSR&D Center for Health Information and Communication, Richard L. Roudebush Veterans Affairs Medical Center, Indianapolis, IN USA; 6 Division of General Internal Medicine & Geriatrics, Indiana University School of Medicine, Indianapolis, IN USA; 7 Regenstrief Institute, Indianapolis, IN USA; 8 Birmingham/Atlanta VA Geriatric Research Education and Clinical Center (GRECC), Department of Veterans Affairs, Birmingham, AL USA; 9 Division of Preventive Medicine, Department of Medicine, University of Alabama at Birmingham, Birmingham, AL USA

## Abstract

**Background:**

Implementation Mapping is an organized method to select implementation strategies. However, there are 73 Expert Recommendations for Implementing Change (ERIC) strategies. Thus, it is difficult for implementation scientists to map all potential strategies to the determinants of their chosen implementation science framework. Prior work using Implementation Mapping employed advisory panels to select implementation strategies. This article presents a data-driven approach to implementation mapping, in which we systematically evaluated all 73 ERIC strategies using the Tailored Implementation for Chronic Diseases (TICD) framework. We illustrate our approach using implementation of risk-aligned bladder cancer surveillance as a case example.

**Methods:**

We developed objectives based on previously collected qualitative data organized by TICD determinants, i.e., what needs to be changed to achieve more risk-aligned surveillance. Next, we evaluated all 73 ERIC strategies, excluding those that were not applicable to our clinical setting. The remaining strategies were mapped to the objectives using data visualization techniques to make sense of the large matrices. Finally, we selected strategies with high impact, based on (1) broad scope, defined as a strategy addressing more than the median number of objectives, (2) requiring low or moderate time commitment from clinical teams, and (3) evidence of effectiveness from the literature.

**Results:**

We identified 63 unique objectives. Of the 73 ERIC strategies, 45 were excluded because they were not applicable to our clinical setting (e.g., not feasible within the confines of the setting, not appropriate for the context). Thus, 28 ERIC strategies were mapped to the 63 objectives. Strategies addressed 0 to 26 objectives (median 10.5). Of the 28 ERIC strategies, 10 required low and 8 moderate time commitments from clinical teams. We selected 9 strategies based on high impact, each with a clearly documented rationale for selection.

**Conclusions:**

We enhanced Implementation Mapping via a data-driven approach to the selection of implementation strategies. Our approach provides a practical method for other implementation scientists to use when selecting implementation strategies and has the advantage of favoring data-driven strategy selection over expert opinion.

**Supplementary Information:**

The online version contains supplementary material available at 10.1186/s13012-022-01231-6.

Contributions to the literature

- Prior work using Implementation Mapping employed advisory panels to select implementation strategies.
- We present a data-driven approach to Implementation Mapping, considering every determinant in the Tailored Implementation for Chronic Diseases (TICD) framework and every Expert Recommendations for Implementing Change (ERIC) strategy.
- We mapped strategies defined by the ERIC to change objectives, using data visualization techniques to make sense of the large matrices created by our comprehensive approach.
- We suggest other implementation scientists use similar techniques in their selection of implementation strategies, favoring data-driven strategy selection over expert opinion.

## Introduction

Implementation mapping has recently been described as an organized way to develop or select implementation strategies through five specific tasks guided by an implementation science framework [1]. The process of selecting implementation strategies can be challenging for implementation scientists. Appropriate strategies are guided by an implementation science theory or framework and consider contextual factors and known implementation barriers, which may differ across key stakeholders such as leaders, nurses, or providers [2]. One specific approach to the selection of implementation strategies is to map strategies to the determinants of the chosen implementation science framework, as initially described in 2019 as part of implementation mapping [1]. Since then, several researchers have reported on their application of implementation mapping. According to these reports, researchers used advisory groups (e.g., task force or stakeholder advisory group) to select implementation strategies from potentially applicable Expert Recommendations for Implementing Change (ERIC) strategies [3, 4]. While this approach worked, selection of strategies likely depended on the composition of these advisory groups and on the opinion of the individuals comprising them. Thus, one potential area for improvement in the application of implementation mapping is the use of a systematic data-driven approach to reviewing and prioritizing all 73 ERIC strategies.

For this reason, we operationalized implementation mapping through a data-driven process, considering all 73 ERIC strategies and every determinant of the Tailored Implementation for Chronic Diseases (TICD) framework. We used data visualization techniques to manage the consequently large number of objectives and ERIC strategies. In this manuscript, we illustrate our data-driven approach to implementation mapping using implementation of risk-aligned bladder cancer surveillance as a case example. Our approach is intended for use by implementation scientists who seek a rigorous selection process for implementation strategies.

### Case example: risk-aligned bladder cancer surveillance

Bladder cancer is one of the most prevalent cancers in the Department of Veterans Affairs (VA) [5]. The vast majority of patients with bladder cancer have early stage cancer, which only grows superficially within the bladder [6]. Early stage bladder cancer patients undergo resection of the cancer from the bladder and are then at varying risks of cancer recurrence within the bladder—categorized as low, intermediate, and high according to current guidelines [7]. To detect these recurrences, patients undergo regular surveillance cystoscopy procedures, during which providers directly inspect the bladder via an endoscope. Given the broad range of cancer recurrence risks, providers should align the frequency with which patients undergo surveillance cystoscopy procedures with each patient’s individual risk of cancer recurrence. However, we previously found that there is both underuse of surveillance among high-risk and overuse of surveillance among low-risk patients, with up to three quarters of low-risk patients undergoing more procedures than are recommended [8]. Thus, we embarked on selecting implementation strategies to promote risk-aligned bladder cancer surveillance using a data-driven approach to implementation mapping.

## Methods

### Overview

**Fig. 1:**
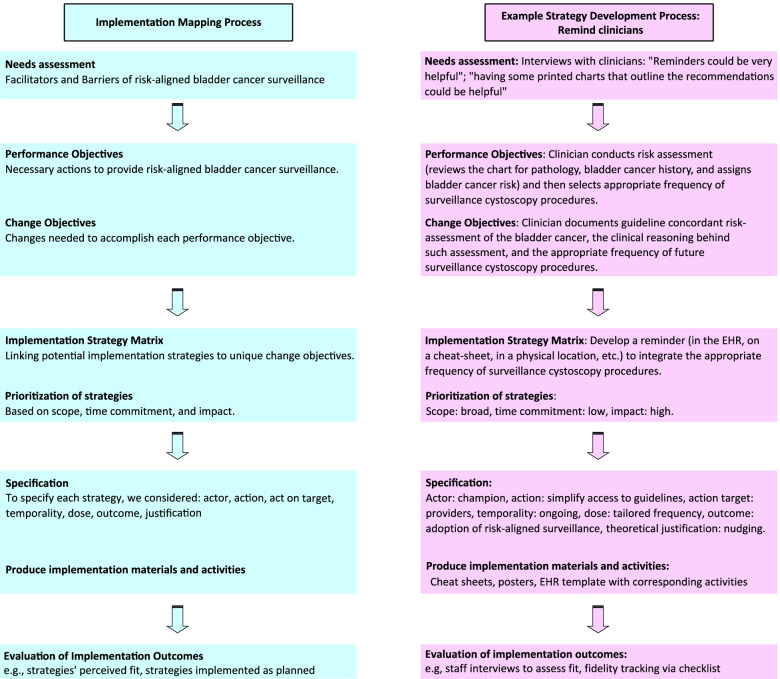
Implementation Mapping process as applied to the current project. The left column shows the specific Implementation Mapping tasks and the right column shows an example strategy that was selected and specified using Implementation Mapping

We employed implementation mapping guided by the TICD framework. Implementation mapping is a systematic process based on five tasks to develop or select strategies for the implementation of evidence-based practice [1]. The TICD framework was chosen because (1) it is an implementation science framework designed to guide efforts to improve care delivery; (2) it is based on a systematic review of 12 prior frameworks; (3) it has been widely used with more than 700 citations in the literature; and (4) it includes a patient factors domain [9]. The TICD includes 57 practice determinants across 7 domains [9]. In the following sections, we describe the implementation mapping tasks used to select and specify implementation strategies for risk-aligned bladder cancer surveillance (Fig. 1). The final task is an ongoing comprehensive evaluation of implementation outcomes to measure the impact of the strategies being pilot tested in four VA sites.

### Needs assessment

The implementation mapping process was based on a needs assessment, for which we identified facilitators and barriers of risk-aligned bladder cancer surveillance. This was done via staff interviews across six Department of Veterans Affairs (VA) sites and has recently been published [10]. In this prior mixed-methods work, we used a quantitative approach to identify the six VA sites. Two sites commonly provided risk-aligned surveillance and four sites were deemed to have room for improvement, defined as sites which performed high intensity surveillance for low-risk and low intensity surveillance for high-risk early stage bladder cancer [10]. We purposively sampled 14 participants (6 providers, 2 nurses, 2 schedulers, 4 leaders) from risk-aligned sites and 26 participants (12 providers, 3 nurses, 3 schedulers, 8 leaders) from sites with room for improvement for semi-structured interviews. In sites with room for improvement, we found that absence of routines to incorporate risk-aligned surveillance into clinical workflow was a salient determinant contributing to less risk-aligned surveillance. Irrespective of site type, we found a lack of knowledge of guideline recommendations by nurses and providers, including attending and resident physicians, and advanced practice providers. We concluded that future implementation strategies will need to address the lack of routines to incorporate risk-aligned surveillance into clinical workflow, potentially via reminders or templates. In addition, implementation strategies addressing knowledge and resources could likely contribute to more risk-aligned surveillance [10].

### Identification of performance and change objectives

This task entailed identification of two types of objectives, performance objectives and change objectives. Performance objectives are *observable actions that need to be performed* to provide risk-aligned bladder cancer surveillance and define “who has to do what” [11]. Change objectives are defined by what needs to be changed related to a specific determinant to accomplish the performance objective [11].

The performance objectives were organized by TICD framework domains and determinants and then by employee type (provider, nurse, scheduler, leader, patient). Performance objectives were formulated based on qualitative data from the prior staff interviews [10] and then reviewed and discussed in group sessions with the research team to assure they align with the qualitative data. These performance objectives were then discussed with one patient advisory group and one physician advisory group to solicit input.

To formulate change objectives, we then created a change matrix. Each row represented a specific performance objective. The columns listed the 57 determinants from the TICD framework [9]. In each cell of the change matrix, we denoted the change objective, i.e., what needs to be changed to accomplish the performance objective. Directionality was taken into account, i.e., the change objective had to logically affect the performance objective. To formulate the change objectives, two authors (AOO or FRS) independently filled in a first objective into applicable cells. Next, they reviewed each others’ work and then met to discuss edits, including addition of change objectives that were not identified on the initial pass, or changing cells to not being related to a performance objective after discussion. The change matrix was then reviewed by the research team and edited until consensus was reached on the content for each cell of the change matrix. From this final change matrix, we then obtained the unique change objectives. The change objectives were then reduced by combining change objectives that had conceptually overlapping topics.

### Selection of implementation strategies

First, we developed an implementation strategy matrix linking unique change objectives (rows) to potential implementation strategies (columns). Implementation strategies were obtained and labeled according to the ERIC [12]. We reviewed all 73 ERIC strategies and excluded those that were not applicable for inclusion in our project (e.g., not feasible within confines or budget of the project, not appropriate for the context of working within VA, already completed as part of the mixed-methods needs assessment or as part of the research project development). Specifically, one author (FRS) performed an initial assessment of which ERIC strategies may not be applicable for inclusion in our project and specified reasons for exclusion. These decisions were then reviewed, discussed, and revised in meetings with two additional authors (AOO, LZ), and then with the entire research team. All decisions were documented along with reasons for exclusion (see methods journal tab in final implementation strategy matrix in Supplementary Material). Next, we wrote strategy-specific statements in each cell of the matrix on how each strategy could potentially affect a change objective. These statements were discussed by the team, and we came to consensus on the content for each cell of the implementation strategy matrix. The potential implementation strategies were then discussed with one patient advisory group and one physician advisory group to solicit input.

To prioritize strategies, we then created a plot from this matrix, showing how many and which change objectives are being addressed by each strategy. We categorized strategies into broad versus narrow scope based on whether or not they addressed eleven or more change objectives. Eleven or more was chosen as a cut-point because the median number of change objectives addressed by the strategies was 10.5. Next, we evaluated 3 factors for each strategy: (1) broad versus narrow scope based on number of change objectives addressed, (2) qualitative assessment of the required time commitment from local staff, and (3) likely impact of the strategy in our clinical setting based on the available evidence from prior studies. When drawing conclusions about likely impact, we specifically considered the clinical setting in which the prior studies were conducted and whether that setting was comparable to the setting of the current study. As a final task, we decided which strategy should be included or excluded, and reasons for inclusion and exclusion were documented along with the theoretical change methods driving each strategy [13].

### Specification and production of implementation materials and activities

This task included operationalization and specification of each implementation strategy according to seven dimensions described by Proctor, including actors, actions, targets of actions, temporality, dose, implementation outcomes affected, and theoretical justification [14]. In addition, we produced implementation materials for each strategy (e.g., cheat sheets, posters, templates for the electronic medical record) with corresponding implementation activities. These were documented, including fidelity measures (i.e., non-modifiable components of each strategy) and allowable adaptations (i.e., allowable modifications based on local needs). Given the iterative nature of implementation mapping, we occasionally readdressed a prior task throughout the mapping process.

## Results

### Identification of performance and change objectives

We identified 49 performance objectives, i.e, *observable actions that need to be performed* to provide risk-aligned bladder cancer surveillance (Supplementary Material). To demonstrate the process from start to finish, Fig. 1 includes an example (right column). In the example, a performance objective is that each clinician conducts a risk assessment and then selects the appropriate frequency of risk-aligned bladder cancer surveillance (Fig. 1). Each performance objective was mapped against the 57 determinants of the TICD framework to develop the change matrix. The full change matrix is shown in the Supplementary Material, and an example is shown in Table 1. A change objective in the example shown in Fig. 1 (right column) is that a clinician documents guideline concordant risk assessment of the bladder cancer, the clinical reasoning behind such assessment, and the appropriate frequency of future surveillance cystoscopy procedures. The full change matrix included 107 unique change objectives in its cells. After combining those with conceptually overlapping objectives, 63 change objectives remained.

**Table 1:**
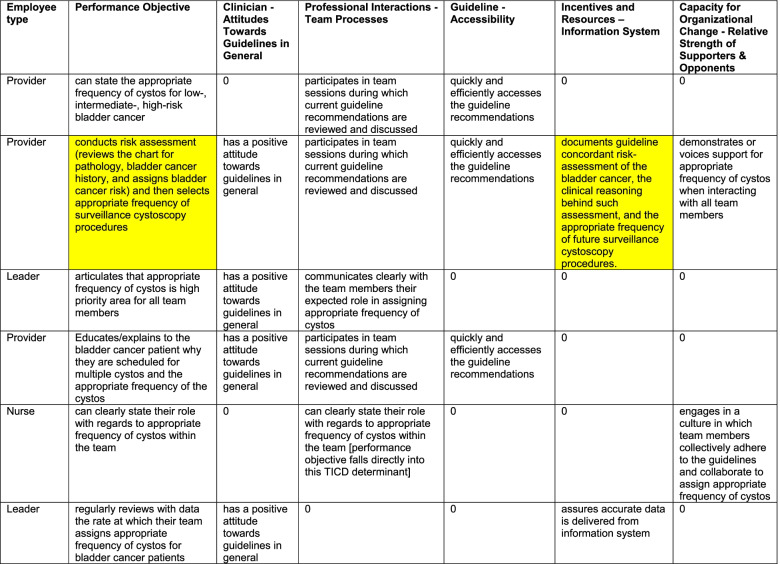
Example of the change matrix

Each row represents a performance objective, i.e., a task that needs to be completed to implement risk-aligned bladder cancer surveillance. Columns 3 through 7 list determinants from the TICD framework. In each applicable cell, we formulated a change objective, defined by what needs to be changed related to a framework determinant to accomplish the task that would lead to more risk-aligned surveillance. Cells that support the example in Fig. 1 are highlighted in yellow

*Cystos* = cystoscopies. A “0” indicates that we did not identify a change objective related to the respective performance objective and TICD determinant. *CME* = continuous medical education

### Selection of implementation strategies

The 63 unique change objectives were mapped against ERIC implementation strategies in the implementation strategy matrix. Of the 73 ERIC strategies, 45 were excluded because they were not applicable to our clinical setting (e.g., not feasible within the confines of the setting, not appropriate for the context, see full implementation strategy matrix in Supplementary Material for documentation of all reasons). Thus, 28 ERIC strategies were mapped to the 63 change objectives within the implementation strategy matrix (Supplementary Material). In Fig. 1 example (right column), an ERIC strategy was development of a reminder to integrate the appropriate frequency of surveillance cystoscopy procedures into routine care, which would make it easier for clinicians to document guideline concordant risk-assessment and surveillance.

**Fig. 2:**
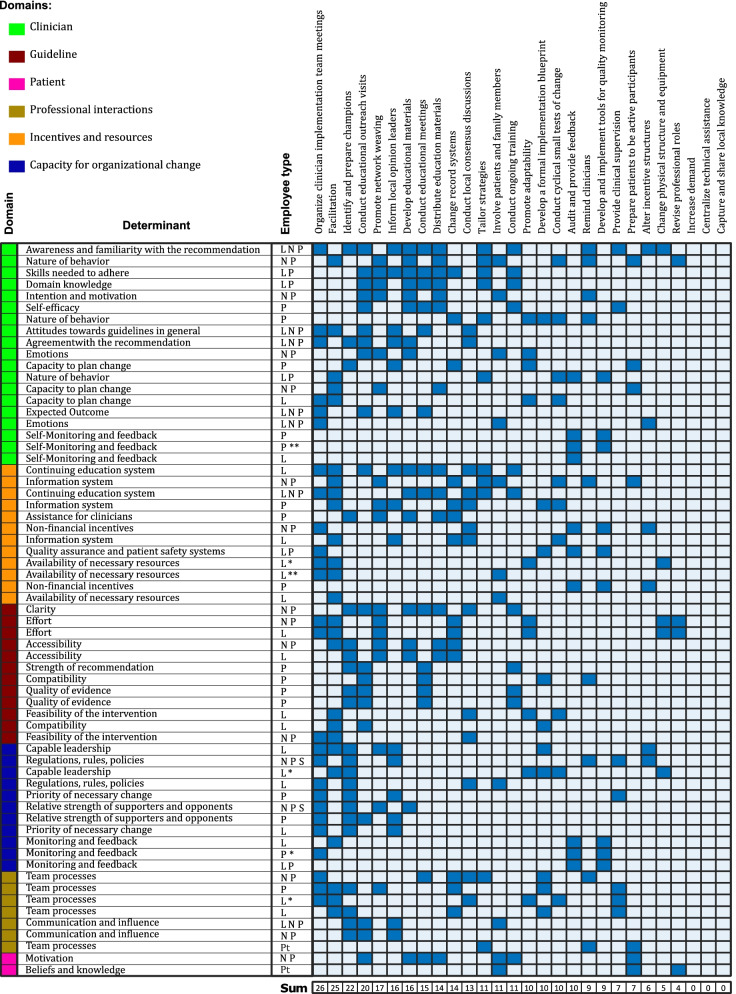
Summary plot of the implementation strategy matrix. Each row represents one of the 63 change objectives listed by TICD determinant along with the employee type who would have to implement the change. Each column represents one of the 28 ERIC strategies that were mapped to the change objectives. If a strategy was classified as affecting a change objective, the cell in the matrix was filled blue. At the bottom of each column, the number of change objectives addressed by each strategy is listed. L = Leader; N = Nurse; P = Provider; * = second assignment for the same determinant – employee type combination; ** = third assignment for the same determinant – employee type combination

**Fig. 3:**
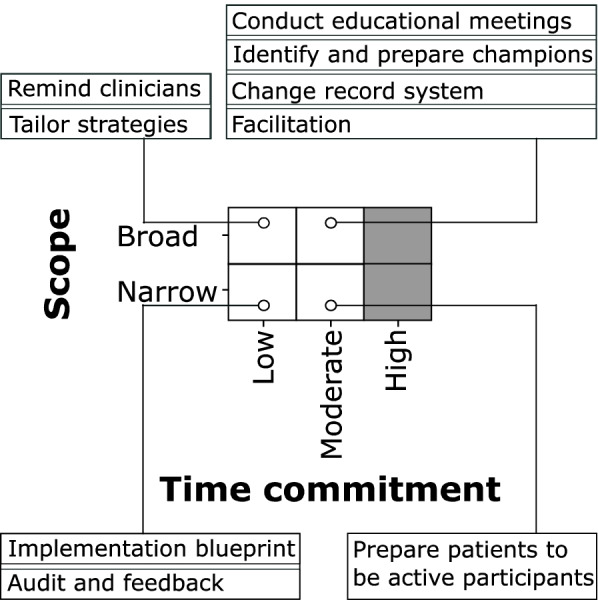
Nine implementation strategies selected based on high impact. To select strategies with high impact, we considered (1) broad versus narrow scope based on number of change objectives addressed, (2) qualitative assessment of the required time commitment from local staff, and (3) likely impact of the strategy in the setting of our project based on the evidence available from prior studies

To better interpret the information contained in the implementation strategy matrix, we created a plot showing how many and which change objectives are being addressed by each strategy (Fig. 2). Each ERIC strategy addressed 0 to 26 change objectives (median 10.5, Fig. 2). Fourteen strategies had a broad scope because they addressed a range of 11 to 26 tasks. Of the 28 ERIC strategies, 10 required low and 8 moderate time commitments from clinical teams. We selected 9 strategies based on high impact (Fig. 3), each with a clearly documented rationale for selection and justification (Table 2).

**Table 2:**
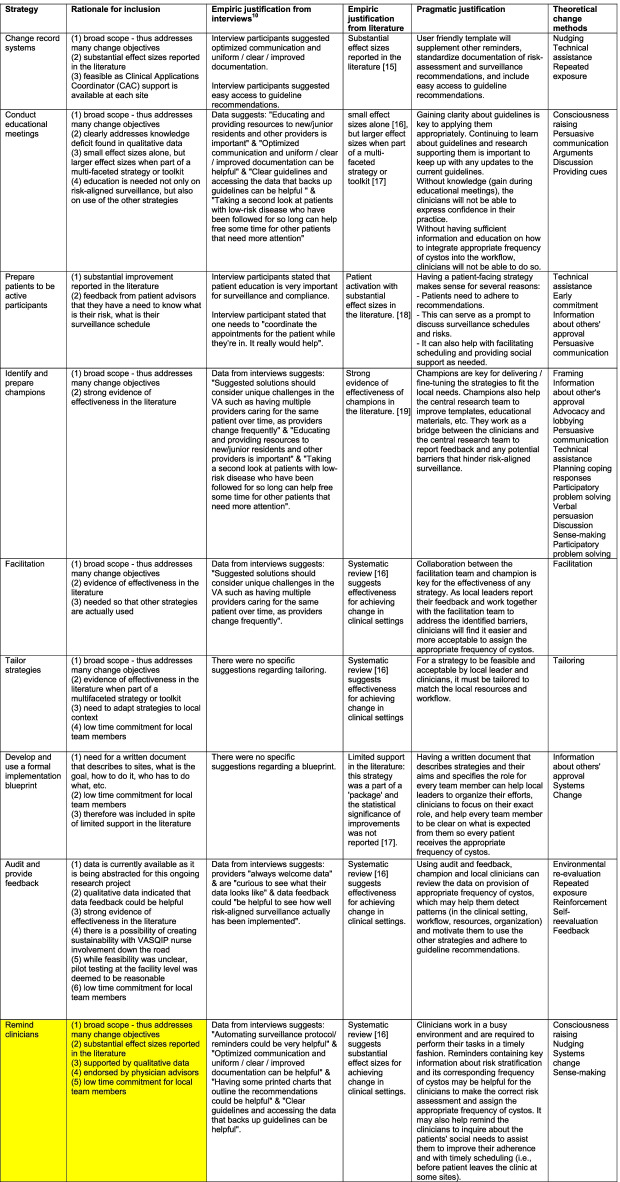
Strategies that were selected along with the rationale and justifications for selection [15–19]

For each strategy, we include an empiric justification from staff interviews [10], an empiric justification from the reviewed literature, and a pragmatic justification formulated by the research team. We also included theoretical change methods likely contributing to the strategies’ desired effects. Cells that support the example in Fig. 1 are highlighted in yellow

### Specification and production of implementation materials and activities

The culmination of the Intervention Mapping process was the production of implementation materials and activities. We used one of the 9 strategies—the implementation blueprint—to codify the remaining 8 strategies for staff members at target sites and guide implementation efforts (Supplementary Material). We noted that there were synergistic effects between strategies, e.g., a local champion will help with educational meetings. Thus, we grouped the 8 strategies into four multifaceted improvement approaches, i.e., groups of implementation strategies that can be delivered together. These included: external facilitation (including facilitation, audit and provide feedback, and tailor strategies), educational meetings (including conduct of educational meetings, and identification and preparation of a champion), reminders (including changing the record system, and reminding clinicians), and prepare patients to be active participants (the only patient-facing improvement approach). The final blueprint included for each improvement approach: (1) what the approach entails, (2) the rationale for the approach, (3) specifics such as location, timing, who needs to do what, (4) a checklist of tasks, (5) expectations regarding minimum number of tasks performed, and (6) space to track any modifications made to the implementation strategies.

## Discussion

We describe a rigorous and data-driven approach to consider every TICD implementation science framework determinant and every ERIC strategy during implementation mapping. We were able to interpret the large matrices by plotting the results of the implementation strategy matrix (Fig. 2) and the factors influencing strategy prioritization and selection (Fig. 3). This rigorous process allowed us to select implementation strategies primarily based on data rather than on opinions of the advisory groups alone. The implementation mapping process culminated in highly specified implementation strategies that were codified in an implementation blueprint.

Our approach is novel as the selection of implementation strategies was driven primarily by data. Prior work using implementation mapping employed advisory panels to select implementation strategies out of potential ERIC strategies [3, 4], which is more subjective, or did not clearly report how the selection was handled [20]. To overcome this limitation, we created an implementation strategy matrix, cross-walking all potentially applicable ERIC strategies against all change objectives. We then developed a plot visualizing this large matrix (Fig. 2). This allowed us to evaluate the scope of each ERIC strategy, based on the change objectives that were addressed. The plot also included visualization of which TICD framework domains and determinants were addressed by each strategy along with which employee types would be involved. This comprehensive representation of all mapping data then drove the decisions of which strategies to select.

To our knowledge, this study is the first to apply implementation mapping as recommended by Fernandez et al. [1] to improve guideline-concordant cancer care delivery in the clinic. Prior studies used implementation mapping in oncology to implement a phone navigation program [21] and exercise clinics in oncology [4], but not yet to directly improve cancer care delivery in the clinic.

We would like to emphasize that our data-driven approach to implementation mapping is not limited to a specific implementation science framework. Whereas our change objectives were categorized by TICD domains and determinants, other frameworks that can guide systematic categorization of determinants of evidence-based practice can be used in similar fashion. For example, the initial description of implementation mapping specifically mentions use of the Consolidated Framework for Implementation Science [22] and the Theoretical Domains Framework [23] as other suitable framework options [1].

It is important to acknowledge issues of equity and stakeholder preferences and values in the selection of implementation strategies. In our data-driven approach, equity and stakeholder preferences were included to the extent that they were represented in the prior mixed-methods assessments of staff needs [10]. However, diversity among stakeholders recruited for interviews and participation in advisory panels was somewhat limited with 8% African American and 2% Hispanic representation among interview participants [10] and no African American representation in our advisory panels. This could be seen as a limitation of our specific work and case example. However, our data-driven approach could easily be adapted for projects focused on diversity, equity, and inclusion. For example, one could use the Health Equity Implementation framework [24] to incorporate equity-relevant determinants into the data-driven implementation mapping process, optimizing the scientific yield and equity of implementation efforts [25].

Despite our approach’s innovation and rigor, there are several limitations to discuss. First, opinions of the research team affected certain parts of the implementation mapping process. This included the assessment of time commitment for local teams as well as the interpretation of the available literature when assessing the overall impact of a strategy. However, we tried to limit subjectivity as much as possible to focused questions and by including different perspectives from an implementation scientist, a urologist, an internist, and several implementation research staff members in this process. Second, whereas our implementation mapping process was primarily driven by data, we did not formally assess its reproducibility by an independent team. Third, the data-driven approach relied mostly on the work of the research team and a formal co-design approach was not included in the selection of the implementation strategies. Fourth, this study was focused on improving cancer surveillance in the VA, so findings regarding the impact of the selected implementation strategies may not readily translate to other healthcare settings or different clinical problems. However, our data-driven approach to implementation mapping will likely be helpful to others regardless of healthcare setting or clinical problem being addressed. Finally, implementation mapping in general is quite labor intensive. Our data-driven implementation mapping took about a year of part-time investigator and full-time research assistant effort. However, we were unable to quantify how much more effort was required for our approach compared to prior studies, as the authors of the prior studies did not report the amount of time, personnel, and expertise needed for their work [1, 20, 21]. We recognize that this level of rigor may not always be possible in our current era of rapid research or during routine operational activities. However, our visualization of the implementation strategy matrix (Fig. 2) could still be integrated into implementation mapping and will likely be helpful for researchers to understand, interpret, and present results.

It is also quite possible that our data-driven approach yielded additional information that otherwise might have been overlooked in implementation mapping as previously applied. Future work could address the empirical question whether our data-driven approach yielded additional information compared to an advisory panel approach, and whether this information is important enough to justify the additional time needed to complete the highly data-driven implementation mapping process.

## Conclusions

In conclusion, we described a data-driven and rigorous implementation mapping process to select implementation strategies for risk-aligned bladder cancer surveillance. The implementation strategies are currently being pilot-tested across four VA sites, with the goal of measuring implementation outcomes and adapting strategies to different local preferences. Once piloting is complete, future work will likely entail testing both the strategies and the clinical innovation (i.e., risk-aligned bladder cancer surveillance) in a larger number of sites. We hope that our work will inspire other implementation scientists to use similar data-driven processes in their selection of implementation strategies, minimizing the risk of bias being introduced by heavy reliance on the opinions of advisory groups.

## Supplementary Information

**Additional file 1.** Final performance objectives.

**Additional file 2.** Final change matrix.

**Additional file 3.** Final Implementation Strategy Matrix.

## Data Availability

All data generated or analyzed during this study are included in this published article and its supplementary information files.

## Abbreviations

cysto: Cystoscopy
ERIC: Expert Recommendations for Implementing Change
TICD: Tailored Implementation for Chronic Diseases
VA: Department of Veterans Affairs
